# Supplementary material for: Development of new real-time PCR assays for detection and species differentiation of Plasmodium ovale
Source: PLoS Negl Trop Dis. 2024 Sep 10;18(9):e0011759. doi: 10.1371/journal.pntd.0011759 (PMC11414980; doi:10.1371/journal.pntd.0011759)
Supplement: S1 Table — (DOCX) [file pntd.0011759.s001.docx]

**S1 Table. Molecular assays to distinguish *P. ovalecurtisi* and *P. ovalewallikeri.***

| Study | Target | Limit of detection | Reaction volume  (µl) | DNA template volume (µl) | Cycles | PCR Reaction time (min) | Reaction step | Comments |
| --- | --- | --- | --- | --- | --- | --- | --- | --- |
| Potlapalli, et al. (2023) | 18S rRNA | 0.1 parasites/µl  (*P. ovalecurtisi*);  0.8 parasites/µl  (*P. ovalewallikeri*) | 50 | 5 | 50 | 75 | Separate real-time PCR reaction for each species | - |
| Oguike, et al. (2011) | PoTRA (Tryptophan-rich  antigen) | NA | - | - | 44 (Primary and secondary amplification) | 106  (Primary and secondary amplification) | Two PCR runs, gel electrophoresis, and sequencing | Determine species by different PCR product fragment size and sequencing |
| Oguike, et al. (2011) | Porbp2 (reticulocyte-binding protein 2) | NA | - | - | 40 | 72 | One real-time PCR run for both species | Determine species by melt-curve analysis |
| Tanom sing, et al. (2013) | PoTRA | 2 to 10 parasites/µl blood for both *P. ovalecurtisi* and *P. ovalewallikeri* | 20 | 1 | 25 (Primary amplification); 30 (Secondary amplification) | 81 (Primary amplification); 96 (Secondary amplification) | Two PCR runs, gel electrophoresis, and sequencing | Determine species by different PCR product fragment size |
| Perandin, et al. (2004) | 18S rRNA | 1.5 parasites/µl for *P. ovalecurtisi* | 50 | 5 | 45 | 68 | One real-time PCR run | Detect *P. ovalecurtisi* |
| Calderaro, et al (2012) | 18S rRNA | 50 target copies for *P. ovalewallikeri* | 50 | 5 | 55 | 81 | One real-time PCR run | Detect *P. ovalewallikeri* |
| Lamien-Meda, et al. (2019) | *Clpc*  (Caseinolytic  protease C) | 8.9 copies/µl  (*P. ovalecurtisi*);  9.61 copies/µl  (*P. ovalewallikeri*) | 20 | 4 | 50 | 80 | A qPCR-HRM assay | Determine species by Tm values |
| Lamien-Meda, et al. (2019) | *Clpc* | NA | 20 | 2 | 50 | 80 | Snapback assay | Determine species by Tm values |
| Nundu, et al. (2021) | *cox3* (cytochrome c oxidase III) | NA | 25 (Primary and secondary amplification) | 5 (Primary amplification); 2 (Secondary amplification) | 40 (Primary and secondary amplification) | 106 (Primary and secondary amplification) | Two PCR runs, gel electrophoresis, and sequencing | Determine species by sequencing |
| Joste, et al. (2018) | 18S rRNA | 1 parasite/µl for both *P. ovalecurtisi* and *P. ovalewallikeri* | 25 | 5 | 40 | 87 | A qPCR-HRM assay | Determine species by Tm values |
| Fuehrer, et al. (2012) | 18S rRNA | NA | 50 (Primary amplification);  25 (Secondary amplification) | 2 (Primary amplification); 2.5 (Secondary amplification) | 40 (Primary amplification);  25/35 (Secondary amplification) | 154 (Primary amplification);  110/150 (Secondary amplification) | Two PCR run, gel electrophoresis | Primary amplification is genus specific; secondary amplification is species specific; determine species by gel electrophoresis |
| Miller, et al. (2015) | Porbp2 | 1.5 copies/µl | 20 | 1 | 40-60 | 23-34 | One real-time PCR run | The *P. ovale* specific real-time PCR assay can determine species by sequencing |
| This study  (Duplex real-time PCR assay) | Putative liver stage antigen 3 (lsa3) gene for *P. ovalecurtisi*; non-coding region on chromosome 14 for *P. ovalewallikeri* | 4.2 parasites/µl (*P. ovalecurtisi*); 41.2 parasites/µl (*P. ovalewallikeri*) | 10 | 3 | 45 | 68 | One real-time PCR run | - |
| This study  (Singleplex real-time PCR assay) | Putative liver stage antigen 3 (lsa3) gene for *P. ovalecurtisi*; non-coding region on chromosome 14 for *P. ovalewallikeri* | 3.6 parasites/µl (*P. ovalecurtisi*); 25.9 parasites/µl (*P. ovalewallikeri*) | 10 | 3 | 40 (*P. ovalecurtisi*); 45 parasites/µl (*P. ovalewallikeri*) | 62 (*P. ovalecurtisi*); 68 (*P. ovalewallikeri*) | Separate real-time PCR reaction for each species | - |
