# Supplementary material for: Development of new real-time PCR assays for detection and species differentiation of Plasmodium ovale
Source: PLoS Negl Trop Dis. 2024 Sep 10;18(9):e0011759. doi: 10.1371/journal.pntd.0011759 (PMC11414980; doi:10.1371/journal.pntd.0011759)
Supplement: S2 Table — (DOCX) [file pntd.0011759.s002.docx]

**S2 Table. Candidate primer and probe sets evaluated for the detection of *P. ovalecurtisi* and *P. ovalewallikeri.***

| Name | Sequence (5’-3’) |
| --- | --- |
| Poc_Fwd1 | GTTRCCAAATATGCTATCACTTAC |
| Poc_Rev1 | GTARCACAAAACGACGAGAC |
| Poc_Probe1 | FAM - TACATCTTCTTCAAAGTTGYCATAYGCAT - BHQ1 |
| Poc_Fwd2 | TTTGTTTGGTGTTTCTTTTTTATCTTC |
| Poc_Rev2 | CCAGAAGAACAATACAATACTATAGA |
| Poc_Probe2 | FAM - TTTCCTTTYATTTCCTCGATTACT - BHQ1 |
| Poc_Fwd3 | CAAATATGCTATCACTTACATCGTTWTG |
| Poc_Rev3 | ATGTARCACAAAACGACGAGACTAAT |
| Poc_Probe3 | FAM - TACATCTTCTTCAAAGTTGYCATAYGCAT - BHQ1 |
| Poc_Fwd4 | TATTGACGAATTGGAAGTWGAMTTRR |
| Poc_Rev4 | CCTWCYAGCTGCACCYTCAGG |
| Poc_Probe4 | FAM - TCMGYGCATTYGATTCMTCGTTCTT - BHQ1 |
| Poc_Fwd5 | CAAATATGCTATCACTTACATCGTTWTG |
| Poc_Rev5 | ATGTARCACAAAACGACGAGACTAAT |
| Poc_Probe5 | FAM - TACATCTTCTTCAAAGTTGYCATAYGCAT - BHQ1 |
| Pow_Fwd1 | GRRTCTTCTGAACTTTGRAATG |
| Pow_Rev1 | CATCAAGGRTATCCATTTCA |
| Pow_Probe1 | VIC - AACAAYCACTTCAACATCAA - BHQ1 |
| Pow_Fwd2 | TGGAGAGGACATWAAGRGARAAG |
| Pow_Rev2 | TTTCCTTTYATTTCCTCGATTACT |
| Pow_Probe2 | VIC - TTKCCTTTTATGTCMRTGT - BHQ1 |
| Pow_Fwd3 | GTTATCGACTCCTTCATTGTT |
| Pow_Rev3 | GTAGAAAACGACATGGCTAATG |
| Pow_Probe3 | VIC - TTCCTCGAAGTCACCAAACACA - BHQ1 |
