# Supplementary material for: Development of new real-time PCR assays for detection and species differentiation of Plasmodium ovale
Source: PLoS Negl Trop Dis. 2024 Sep 10;18(9):e0011759. doi: 10.1371/journal.pntd.0011759 (PMC11414980; doi:10.1371/journal.pntd.0011759)
Supplement: S3 Table — (DOCX) [file pntd.0011759.s003.docx]

**S3 Table. Sequences contained in synthetic plasmids to determine assay analytical sensitivity.**

| Species | Sequence (5’-3’) |
| --- | --- |
| *P. ovalewallikeri* | TTGAAATGGATCTTCTGAACTTTGGAATGGATATCCTTGATGTTGAAGTGGTTGTTCAGAACTTTGAAATGGATATCCTTGATG |
| *P. ovalecurtisi* | GTTGCCAAATATGCTATCACTTACATCGTTTTGTGCTACATCTTCTTCAAAGTTGTCATATGCATTATTAGTCTCGTCGTTTTGTGCTACAT |
