# Supplementary material for: Development of new real-time PCR assays for detection and species differentiation of Plasmodium ovale
Source: PLoS Negl Trop Dis. 2024 Sep 10;18(9):e0011759. doi: 10.1371/journal.pntd.0011759 (PMC11414980; doi:10.1371/journal.pntd.0011759)
Supplement: S4 Table — Parasite density = plasmid DNA copy number/copy number of the target in the parasite genome. (DOCX) [file pntd.0011759.s004.docx]

**S4 Table. Limit of detection of the optimized, singleplex *P. ovalecurtisi* and *P. ovalewallikeri* assays versus serially diluted plasmid DNA.**

| Species | Parasite density (parasites/µl) | Positive (n) | Replicates tested (n) | Proportion detected (%) |
| --- | --- | --- | --- | --- |
| *P. ovalecurtisi* | 10,000 | 3 | 3 | 100 |
| *P. ovalecurtisi* | 1,000 | 3 | 3 | 100 |
| *P. ovalecurtisi* | 100 | 3 | 3 | 100 |
| *P. ovalecurtisi* | 10 | 20 | 20 | 100 |
| *P. ovalecurtisi* | 5 | 19 | 20 | 95 |
| *P. ovalecurtisi* | 2.5 | 19 | 20 | 95 |
| *P. ovalecurtisi* | 1.25 | 12 | 20 | 60 |
| *P. ovalecurtisi* | 1 | 14 | 20 | 70 |
| *P. ovalecurtisi* | 0.1 | 4 | 20 | 20 |
| *P. ovalewallikeri* | 10,000 | 3 | 3 | 100 |
| *P. ovalewallikeri* | 1,000 | 3 | 3 | 100 |
| *P. ovalewallikeri* | 100 | 20 | 20 | 100 |
| *P. ovalewallikeri* | 80 | 20 | 20 | 100 |
| *P. ovalewallikeri* | 50 | 20 | 20 | 100 |
| *P. ovalewallikeri* | 40 | 20 | 20 | 100 |
| *P. ovalewallikeri* | 25 | 19 | 20 | 95 |
| *P. ovalewallikeri* | 12.5 | 4 | 20 | 20 |
| *P. ovalewallikeri* | 10 | 7 | 20 | 35 |
| *P. ovalewallikeri* | 5 | 2 | 20 | 10 |
| *P. ovalewallikeri* | 1 | 0 | 20 | 0 |
