# Supplementary figures and images for: Development of new real-time PCR assays for detection and species differentiation of Plasmodium ovale
Source: PLoS Negl Trop Dis. 2024 Sep 10;18(9):e0011759. doi: 10.1371/journal.pntd.0011759 (PMC11414980; doi:10.1371/journal.pntd.0011759)

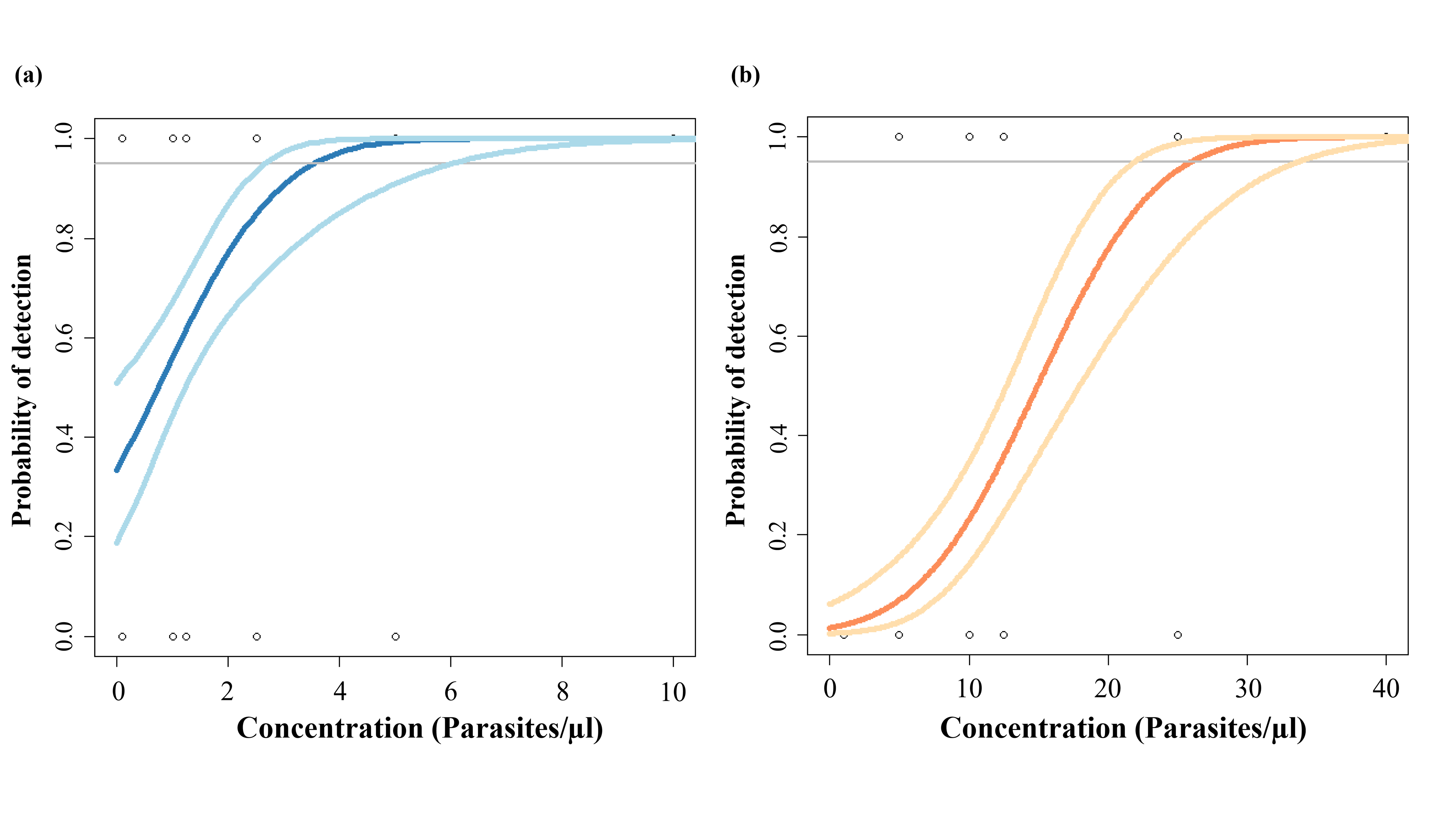

Supplement: S1 Fig — A) P. ovalecurtisi singleplex assay 95% lower limit of detection (3.6 parasites/μl [95% CI 2.7–6]). B) P. ovalewallikeri singleplex assay 95% lower limit of detection (25.9 parasites/μl [95% CI 22–33.6]). Confidence intervals are shown in lighter shade. (TIF) [file pntd.0011759.s006.tif]

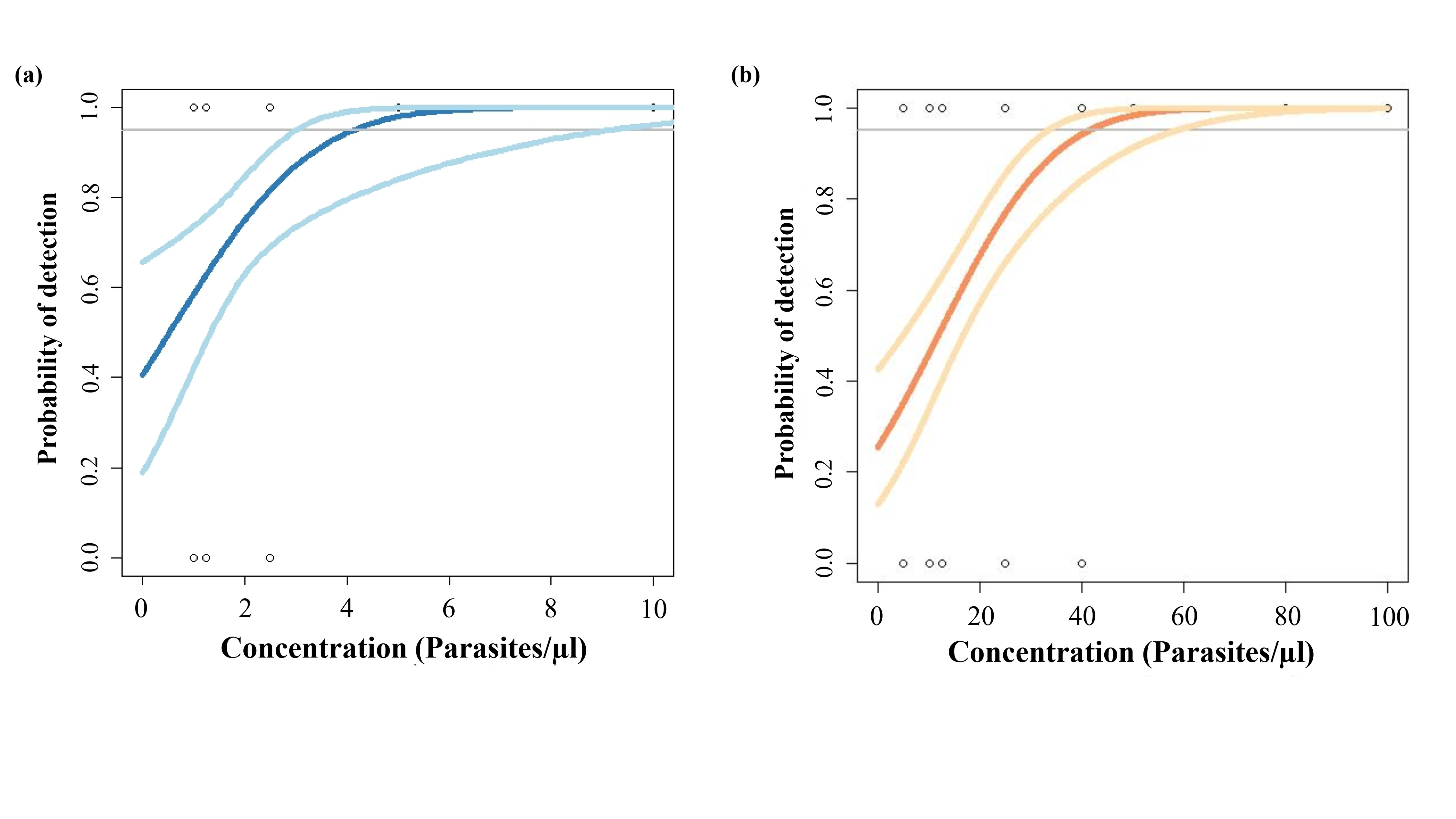

Supplement: S2 Fig — A) P. ovalecurtisi 95% lower limit of detection (4.2 parasites/μl [95% CI 3.1–9.5]). B) P. ovalewallikeri 95% lower limit of detection (41.2 parasites/μl [95% CI 33.3–58.3]). Confidence intervals are shown in lighter shade. (TIF) [file pntd.0011759.s007.tif]
