# Supplementary material for: Development of new real-time PCR assays for detection and species differentiation of Plasmodium ovale
Source: PLoS Negl Trop Dis. 2024 Sep 10;18(9):e0011759. doi: 10.1371/journal.pntd.0011759 (PMC11414980; doi:10.1371/journal.pntd.0011759)
Supplement: Attachment — Submitted filename: review_PNTD-D-23-01354.docx [file pntd.0011759.s008.docx]

Dear editor,

Please find below, my review of the manuscript PNTD-D-23-01354, titled: “A novel duplex qualitative real-time PCR assay for the detection and differentiation of *Plasmodium ovale curtisi* and *Plasmodium ovale wallikeri*” submitted to PLOS Neglected Tropical Diseases.

The authors describe a newly developed duplex real-time PCR assay for the detection and identification of the *Plasmodium ovale* spp. It is a further development of their previous work Potlapalli et al 2023. They detailed their methodology for the selection of the targets, the development and optimization of the assay, the evaluation of its performance on controls, its validation on a set of field samples, and then its application to study the epidemiology of the *Plasmodium ovale* spp. among symptomatic patients in 3 regions of the Democratic Republic of Congo. The manuscript is sounded, well developed and nicely written presenting all the expected steps for such topic.

While this tool will remain of little interest for the Medical doctors, who are still considering, managing and treating both species as one sole entity; this new assay will be useful for the Malariologists and the Malaria elimination program Managers to study the *P. ovale* spp. that are sympatric, wide spread, co-circulating in the endemic areas and widely overlooked. I am glad and would like to thank the authors for their contributing work on these neglected parasites.

Although a couple of points, listed below might need some clarifications / amendments to enhance the manuscript:

- Regarding the nomenclature of the *P. ovale* spp., just recently, a modification in the taxonomy of these species have been proposed (Snounou et al 2024). This clarifies and simplifies the situation and should be adopted throughout the manuscript.
- L24 and L25 : “*P. ovale*”, *Plasmodium* should be written in full as first mention.
- Throughout the manuscript: the use of italic or not for the mentions of “non-*ovale*” and “non-*falciparum*” should be harmonized.
- L129-133: The *Poc* and *Pow* field samples used as controls were “well characterized”, using the earlier protocol proposed by the authors [9], why not comparing this new assay vs their previous one for Poc / Pow distinction and on their practicality and operation to highlight pros and cons?
- L163: “species” no italic
- L167-169: “Samples with ct values lower than 45 in duplicate…” this positivity cut-off sound not compatible with the number of cycles of the PCR which is also 45 (L233).
- L233-234: please precise on which equipment was the PCR done, based on the Fig 1, once can guess QuanStudio 6?
- An Internal Control, either multiplexed in or run in parallel of the developed PCR, would be useful to assess inhibition and/or DNA degradation as well as PCR efficiency. This could also contribute to understand the difference into the sensitivity of the assays.
- L276-294 and Fig 4: conversely to what is advocated across rest of the manuscript, in the epidemiological study of symptomatic malaria cases due to *P. ovale* in 3 regions of DRC, the authors analysed and presented their results on *P. ovale* as a whole entity while being able to discriminate *Poc* and *Pow*. This appears defeating the rest of the manuscript and it would sound better to mention that there was no difference between *Poc* and *Pow* in relation to the epidemiological parameter measured instead of looping both together.
- L328-329: please check and correct this sentence, actually the 18S rRNA are roughly present in the same range of copy number per genome than the target used here in the duplex PCR, this might explain the similar LODs mentioned by the authors (L330-331). Please also amend accordingly below.
- L340: reference [44] is unrelated here.
- L358: “higher” instead of “high”; interestingly in [47] they reported a difference in the distribution of *Poc* between urban and rural areas which was not observed for *Pow*… any similar findings here?
- L386: “eradication strategies” sounds somehow utopic, the best that could be achieved will be elimination.

As a separate though, from a quick glance at the primers and probes sequences, there is likely a substantial gap in the Tm between the *Poc* and *Pow* assay. The authors partly compensated this point during optimization by adjusting the concentration of the different components. But were the assays optimized in singleplex before being combined in duplex? This might give some indications to explain the difference into the assay sensitivity.

Another point to discuss for perspective could be the possibility to convert this assay into a dPCR assay to compensate the difference in sensitivity.

Last, acknowledging the existence of some simian *ovale*-like parasites reservoir, the usefulness and interest of this new assay could be further discussed.

Altogether, it is an interesting manuscript that I would recommend for publication into the PLoS NTD provided some minor amendments are performed.
