# Supplementary material for: Development of new real-time PCR assays for detection and species differentiation of Plasmodium ovale
Source: PLoS Negl Trop Dis. 2024 Sep 10;18(9):e0011759. doi: 10.1371/journal.pntd.0011759 (PMC11414980; doi:10.1371/journal.pntd.0011759)
Supplement: Attachment — Submitted filename: comments from reviewers.docx [file pntd.0011759.s009.docx]

From Editor:

**I concur with the comments of the three reviewers. The concerns of reviewers 1 and 3 with respect to the lower sensitivity for *P. ovalewallikeri* is particularly important: 40 P/µL is very close to the limit of detection by routine thick smear examination, and this is likely to lead to false negative results for many of the infections found in the field, especially in asymptomatic individuals. These two reviewers also suggest that the central claim that the assay represents an improvement on other current molecular assays is not justified. The significance of the epidemiological analyses has also been questioned by these two reviewers.**

**I suggest that significant major amendments are made to any revised manuscript in response to the detailed comments made by the reviewers.**

**Response:** Thanks for this feedback. We agree that our *P. ovale curtisi* assay performs better than the *P. ovale wallikeri* assay, and have attempted to make this clearer in the revised manuscript. We compared the performance of the best performing *P. ovale curtisi* assay when performed singleplex vs. in a duplex format with the *P. ovale wallikeri* assay. We did not observe a loss of sensitivity or specificity when using the duplexed format. We have reframed the manuscript to clearly state use cases – including for epidemiological analyses of *P. ovale curtisi* given the stronger performance of this assay. We also provide clear use cases for the *P ovale wallikeri* assay (whether singleplex or duplex). These additional experiments and specifically the revised discussion have strengthened the manuscript. All line numbers below refer to the line numbers in the tracked version.

**Reviewer's Responses to Questions**

**As you describe the new analyses required for acceptance, please consider the following:**

**Methods**

**-Are the objectives of the study clearly articulated with a clear testable hypothesis stated?**

**-Is the study design appropriate to address the stated objectives?**

**-Is the population clearly described and appropriate for the hypothesis being tested?**

**-Is the sample size sufficient to ensure adequate power to address the hypothesis being tested?**

**-Were correct statistical analysis used to support conclusions?**

**-Are there concerns about ethical or regulatory requirements being met?**

**Reviewer #1: (No Response)**

**Reviewer #2: See attachment**

**Reviewer #3: The objectives and study design are appropriate. The sample size for the validation of the novel assay is appropriate, though limited to geographically restricted samples, while that for the epidemiological analyses is not adequate.**

**Response:** Thanks for the comments. We deleted the epidemiological analysis section in the revised version.

**Results**

**-Does the analysis presented match the analysis plan?**

**-Are the results clearly and completely presented?**

**-Are the figures (Tables, Images) of sufficient quality for clarity?**

**Reviewer #1: (No Response)**

**Reviewer #2: See attachment**

**Reviewer #3: The results and analyses are clearly presented, as are the figures**

**Response:** Thanks for the comments.

**Conclusions**

**-Are the conclusions supported by the data presented?**

**-Are the limitations of analysis clearly described?**

**-Do the authors discuss how these data can be helpful to advance our understanding of the topic under study?**

**-Is public health relevance addressed?</br></br>**

**Reviewer #1: (No Response)**

**Reviewer #2: See attachment**

**Reviewer #3: The limitations listed by the authors are sufficient to weaken some of the conclusions presented and the relevance of the new assay to public health.**

**Response:** Thank you. See comments above and below about differences in performance between the *P. ovale curtisi* and *P. ovale wallikeri* assays, and revised language to make these differences (and potential use cases) clearer.

**Editorial and Data Presentation Modifications? Use this section for editorial suggestions as well as relatively minor modifications of existing data that would enhance clarity. If the only modifications needed are minor and/or editorial, you may wish to recommend “Minor Revision” or “Accept”.**

**Reviewer #1: (No Response)**

**Reviewer #2: See attachment**

**Reviewer #3: Minor comments**

**Response:** Thanks for the comments.

**Summary and General Comments**

**Use this section to provide overall comments, discuss strengths/weaknesses of the study, novelty, significance, general execution and scholarship. You may also include additional comments for the author, including concerns about dual publication, research ethics, or publication ethics. If requesting major revision, please articulate the new experiments that are needed.**

**Reviewer #1:**

**In this simple report, He and colleagues describe an RTqPCR assay for the detection and differentiation of *Plasmodium ovalecurtisi* and *P. ovalewallikeri*. They argue that their assay is ‘less complex’ and ‘streamlined’ compared to other assays, and advocate its use in field studies.**

**Major points.**

1. **Given that their assay is of equal or less sensitivity than other previously reported assays, it is difficult to see the circumstances in which it may replace those currently in use. This assay can only be used in well-funded laboratories with access to real time PCR facilities, and such laboratories would presumably fair better using the more sensitive current methodologies. Of particular concern is the fact that their *P. ovalewallikeri* assay is ten times less sensitive than their *P. ovalecurtisi* assay, rendering it, in my opinion, useless in its ability to assay the relative prevalence of the two species, or, indeed, to identify P*. ovalecurtisi* in relatively low parasite density infections. The authors state in their discussion that their ‘assays sensitivity at lower parasite densities could be further improved’ (line 381). If it can be further improved, then it is perhaps better to do so before publishing and advocating its use.**

**Response:** Thanks for the comments. We regret that the performance of the *P. ovale wallikeri* assay is not equal to that of the *P. ovale curtisi* assay. We agree that this limits our ability to evaluate the relative prevalence of the two species (see Discussion, L471-474). Nonetheless, our assay offers several advantages over most published assays (now detailed in new Table S1), including reduced cycling time, high specificity, and utility for high-throughput use (see Discussion, L407-416, L432-434, and L448-453). The new table shows that most existing assays require prolonged cycling time, nested assays, larger volume of input DNA solution, or both (see Discussion, L398-402).

We do not agree with the reviewer that the difference in performance between *P. ovale curtisi* and *P. ovale wallikeri* renders the duplex assay useless. We performed additional experiments showing that the singleplex and duplex formats of the *P. ovale curtisi* assay have equivalent sensitivity and specificity (see Materials and Methods, L185-191 and L202-205; Results, L293-308 and L322-324). Thus, for *P. ovale curtisi*-focused analyses, an end user can choose their preferred format based on their project’s needs. For example, it could be used as a singleplex *P. ovale curtisi* assay if the goal is to study this species alone, as a duplex assay to identify *P. ovale curtisi* or *P. ovale wallikeri* samples with sufficient DNA concentrations for whole-genome sequencing, or as a duplex assay to quickly assess for the presence of either parasite species (see Discussion, L409-416, L448-453, and L493-497). We also believe there is value in disseminating information about these previously undescribed repetitive targets.

We have added text to the discussion to do a better job of highlighting the assay’s strengths and weaknesses, highlight its relative performance compared to published assays, and provide example use cases.

2. **My second major concern is that the assay was developed using only sequences from a limited region of central Africa. It is possible, therefore, that it may not be suitable for use outside that region, especially the *P. ovalecurtisi* assay, which targets a non-coding region (and so is potentially polymorphic). It would be nice to see some attempt to address this, either just through analysis of polymorphism of the targets in a more diverse pool of *P. ovale* parasites, or through wet lab testing of isolates from outside central Africa.**

**Response:** Thanks for the comments. As part of a separate project, our group used hybrid capture and selective whole genome amplification to whole-genome sequence 13 *P. ovale curtisi* samples from 4 countries (the DRC, Camerron, Tanzania, and Ethiopia) and 12 *P. ovale wallikeri* samples from 3 countries (the DRC, Tanzania, and Ethiopia). We found only low level of variation in the primer or probe binding sites in this unpublished data, nor in publicly available *P. ovale curtisi* and *P. ovale wallikeri* genomes. These results indicate that our assay is suitable for samples from other regions. See pre-print link and further discussion in our response to Reviewer 3 comment #4 below.

**Other points**

3. **Nomenclature. Throughout the manuscript, the two *ovale* species are referred to either as “*P. ovale curtisi*” and ”*P. ovale wallikeri*” or as “*Poc*” and “*Pow*”. It has recently been suggested that the names “*P. ovalecurtisi*” and “*P. ovalewallikeri*” are preferable, as binomials reflect the species nature of the two parasites. I see no need to abbreviate to ‘*Poc*’ and ‘*Pow*’ (we don’t call *P. falciparum* “*P. fal*”, or *P. malariae* “*P. ma*l”). I suggest correcting to “*P. ovalecurtisi*” and “*P. ovalewallikeri*”.**

**Response:** Thanks for the comments. Snounou et al. proposed new nomenclature of the *P. ovale* spp. (Snounou et al., 2024), whereas Šlapeta J et al. (Šlapeta J et al., 2024) raised objections to these new names. Due to the inconsistencies in the nomenclature of the *P. ovale* spp., we use “*P. ovale curtisi*” and “*P. ovale wallikeri*” throughout the revised manuscript. We also acknowledge the new names proposed by Snounou et al. (2024) in the Introduction (see Introduction, L99-101).

4. **Line 25. ‘Species’ are, by definition, ‘non-recombining’. There is no need to add this phrase before the word ‘species’.**

**Response:** Thanks for the comments. We deleted “non-recombining” in the revised manuscript (see Abstract, L24-26).

5. **Line 59. The evidence for this isn’t convincing at the moment, and is somewhat contradictory.**

**Response:** Thanks for the comments. We revised this sentence (see Author Summary, L74-78). Only a limited number of existing assays are able to detect and distinguish *P. ovale curtisi* and *P. ovale wallikeri* without sequencing. Most existing assays target the 18S rRNA gene of both *P. ovale curtisi* and *P. ovale wallikeri*, leading to potential cross-reactivity and a lack of complete species specificity. Some require separate PCR runs, multiple steps or prolonged cycling time that increases risk of false-positive results, hindering our understanding of *P. ovale curtisi* and *P. ovale wallikeri* epidemiology.

6. **Line 71. I’m not sure ‘most malaria programes’ prioritise *P. falciparum*… which programmes are these? Elimination programmes? Control programmes? *P. vivax* is the major issue outside Africa. Perhaps it’s best to specify ‘malaria control programmes within Africa’?**

**Response:** Thanks for the comments. We revised this sentence as suggested (see Introduction, L93).

7. **Line 77 the phrase ‘****distinct non-recombing species’ can be reduced to just ‘species’.**

**Response:** Thanks for the comments. We deleted the “distinct non-recombing” in the revised manuscript (see Introduction, L99-101).

8. **Line 80. There are no single step assays, surely?**

**Response:** Thanks for the comments. We didn’t make it clear in the previous manuscript. There are singleplex real-time PCR assays which need separate reactions for *P. ovale curtisi* and *P. ovale wallikeri* detection (see new Table S1). Other published conventional PCR assays need agarose gel electrophoresis and sequencing for confirmation. We edited this in the revised manuscript (see Introduction, L103-105).

9. **Line 83. What do the authors mean by ‘conventional malaria diagnostic assays relying on microscopy’. Do they mean simply ‘microscopy’?**

**Response:** Thanks for the comments. Detection and differentiation of *P. ovale curtisi* and *P. ovale wallikeri* is not currently possible using microscopy or rapid diagnostic tests. We revised this sentence for clarity (see Introduction, L106-107).

10. **Line 98. ‘this assay requires multiple steps’. What does this mean? ALL assays require multiple steps… And a ‘long turnaround time’? Perhaps it would be helpful if a table is included comparing the various assays for detection and discrimination of the two species. The table could include the sensitivities, the costs, the number of ‘steps’ etc. It would be nice to include the methodology of Nundu et al in reference 47, who performed a simple nested PCR then sequenced the P. ovale positives.**

**Response:** Thanks for the comments. We edited this sentence in the revised manuscript for clarity (see Introduction, L124-127). We added the description of Nundu’s assay in the revised manuscript (see Introduction, L127-129). A table summary of published assays is also included in the revised manuscript (Table S1).

11. **Line 105. Perhaps the authors could explain here what a ‘duplex qualitative real-time PCR assay’ is for the general reader.**

Response: Thanks for the comments. Our duplex assay simultaneously amplifies *P. ovale curtisi* and *P. ovale wallikeri* DNA in a single reaction tube, and it is suitable for the confirmation of the presence of *P. ovale curtisi* and *P. ovale wallikeri.* We did not attempt to develop a quantitative assay (the short distances between repetitive motifs are expected to reduce PCR efficiency), but rather a qualitative (positive/negative) assay. We included this in the revised manuscript (see Materials and Methods, L191-193).

12. **Line 129 What does the term ‘****well-characterised’ mean here?**

**Response:** Thanks for the comments. The “well-characterised” means *Plasmodium ovale* species was determined in these field samples using a different, published approach (see Potlapalli et al. 2023) (Materials and Methods, L171-174).

13. **Line 152. What constitutes a ‘replicate’ in this context?**

**Response:** Thanks for the comment. These are technical replicates of serially diluted *P. ovale curtisi* and *P. ovale wallikeri* plasmid DNA for analytical sensitivity determination. We included the details of the *P. ovale curtisi* and *P. ovale wallikeri* plasmid concentrations and number of the replicates in the revised manuscript (see Tables S4 and S5; Materials and Methods, L202-206).

14. **Line 154 What does the term ‘well-characterized’ mean here?**

**Response:** See response to comment #12 above.

15. **Line 187. Does this mean that of the 64 previously *P. ovale* positive samples, only 44 were found to be positive on repeated analysis? This is quite a significant result – could the authors speculate as to the reason of this significant discrepancy?**

**Response:** We only tested a subset of 44 samples that had previously tested positive in duplicate. Note that we have removed the epidemiological analysis section in the revised manuscript (Materials and Methods, L228-251; Results, L367-390; Discussion, L455-470).

16. **Line 232. It is specified that 3 uL of DNA template was used in the assay. Could the authors explain how much total blood volume this is** **equivalent to? How much blood was used for DNA extraction? Without this data, it is impossible to assess the actual sensitivity of the assay.**

**Response:** In this study, most of the field samples were dried blood spot samples. Each DBS spot typically contains approximately 70µl of whole blood. DNA is extracted from each ~70µl spot and eluted into 150µl final volume. Thus, 3µl of DNA template is roughly equivalent to 1.4µl of whole blood. We included the volume of the whole blood in the revised manuscript (see Materials and Methods, L176-179; Results, L299-300).

17. **Line 239, as above. ‘equivalents/uL’… uL of what? Blood or DNA solution? The approximate volume of blood assayed in each reaction needs to be given.**

**Response:** This refers to µl of DNA template. We have added this to the Results section. We used serially diluted plasmid DNA solution for analytical sensitivity analysis, we included this in the revised manuscript (see Results, L322-326). The blood volume equivalent to “3µl of DNA template” depends on the DNA extraction method. See comments above.

18. **Line 242. What was the negative control for these assays? Was it uninfected blood extracted in the same way as the samples?**

**Response:** We included six negative controls (two *P. falciparum*, one *P. malariae*, two *P. vivax*, and one *P. knowlesi* samples) from an external quality assurance program during assay development. These were dried blood spot samples. DNA in these samples was extracted using Chelex 100. We also included non-template control (nuclease-free water) in each real-time PCR run. These details are provided in the revised manuscript (see Materials and Methods, L174-179, L198-200, and L207-211).

19. **Line 255 is rather an odd sentence. It is in effect saying that sensitivity was excellent when there was lots of parasite DNA present. That’s not a good measure of ‘sensitivity’.**

**Response:** We provide precise estimates of analytical and clinical sensitivity in the Results. We included the information referenced by the reviewer because it can be relevant clinically and when considering study design. For example, symptomatic patients are expected to have higher parasite densities (in contrast to asymptomatic patients). We expect the duplex assay to perform well in these settings. To avoid confusion, we removed this language from the Results and have tried to be clearer elsewhere in the manuscript. (see Results, L346-348; Discussion, L407-416).

20. **Line 258: the number of parasites per ‘uL’ is given again – it is essential to know whether this is uL of DNA solution or of original blood.**

**Response:** This is DNA solution. See comments above. This has been addressed in the revised manuscript (see Results, L349).

21. **Line 259 –** **the assays sensitivity of 80% is compared to what? Blood smear positive? Previous qPCR results?**

Response: The assay sensitivity of 80% is compared to the previously published real-time PCR results using a different assay (Mitchell et al., 2021). We have clarified this point in the revised manuscript (see Materials and Methods, L223-226). All false-negative results occurred in low density *P. ovale* samples (<10 parasites/µl) (see Results, L352-353).

22. **Line 267. The 64 *P. ovale* spp positive samples had been determined as positive using a previous assay, I think. The authors could only confirm 44 of these to be truly *P. ovale* positive using the same assay… if I’ve got this right, then the epidemiological analysis should only be performed on the 44 confirmed *P. ovale* ceases, surely?**

**Response:** See response to comment #15 above. The epidemiological analysis has been removed from the revised manuscript.

23. **Line 267 onwards. The epidemiology section seems somewhat shoe-horned into the paper, and doesn’t really fit with the rest of it – as the risk factor calculations were not split into *P. ovale wallikeri* and *P. ovale curtisi*, this analysis does not depend on the new assay under discussion, and is based on a previously carried out analysis, it seems superfluous to the paper as a whole. It would be much better to just give the breakdown of the two species compositions in the original sample set. Related to this, I fail to see the significance of the performance of an ‘inverted probability weighting analysis’ to extrapolate from 37 samples to 44 (?).**

**Response:** In response to reviewer comments, we have deleted the epidemiological analysis section in the revised manuscripts.

24. **Line 301. The authors describe their assay as “highly specific’. Yet, the *P. ovale curtisi* component is 10 times more sensitive than the Pow component. I can’t reconcile these two statements. They state it can be used to ‘improve our understanding of their epidemiology in malaria-endemic countries’ yet it is less sensitive than other RTqPCR assays and conventional nested PCR? How will it, therefore, improve our understanding?**

**Response:** See responses above. We have attempted to better outline strengths and weaknesses of the assays and provide potential use cases in the revised manuscript (see Discussion, L407-416, L432-434, L452-453, and L471-490).

25. **Line 305 “distinct, non-recombining species” This suggests that there are indistinct, recombining species in existence….**

**Response:** We revised this sentence accordingly (see Discussion, L397-398).

26. **Line 307. Why are higher volumes of DNA required in other assays? The author’s assay is less sensitive, so would require a larger volume of DNA to reach the same level of sensitivity.**

**Response:** See Table S1 for details of other published assays, which were optimized using higher reaction volumes. We have included the input volume for each assay. Details of why specific reaction conditions were selected can be found in the referenced published manuscripts.

27. **Line 308. ‘multiple steps’ is vague. Again, a table comparing the methods alluded to with the authors’ assay would be useful.**

**Response:** This has been addressed above, with inclusion of a new table S1 as requested. We have revised this sentence (see Discussion, L398-402).

28. **Line 319. In world terms, the samples weren’t collected from particularly ‘geographically diverse sites’, all of them being in** **central Africa.**

**Response:** We agree with the reviewer that samples from two countries are not globally representative. However, inclusion of samples from multiple countries nonetheless improves generalizability. Clinical samples were collected in sites spanning thousands of kilometers across one of the most malarious regions of the world. See comments above/below about comparison to newly analyzed genomes from other countries, sequenced as part of a separate project.

29. **Line 325. Could the ‘advantageous performance characteristics’ be given here?**

**Response:** Thanks for the comments. We have revised this sentence and provided more detail as requested (see Discussion, L407-416, L432-434, and L448-453).

30. **Line 345. How were these prevalences determined?**

**Response:** As noted above, the epidemiological analysis section is not included in the revised manuscript.

31. **Line 354 “Existing evidence indicates that the most prevalent *P. ovale* spp. vary across different countries’. Could the authors rewrite this sentence for clarity…?**

**Response:** The epidemiological analysis section was removed from the revised manuscript.

32. **Line 356. What does ‘Poc more prevalent in symptomatic individuals’ mean? More prevalent than in asymptomatic individuals? More prevalent than Pow in symptomatic individuals? If the latter, then this will always appear to be the case when performing the authors' assay, as the Poc sensitivity is 10 times that of Pow.**

**Response:** The epidemiological analysis section was removed from the revised manuscript.

**Reviewer #2:**

**Dear editor,**

**Please find below, my review of the manuscript PNTD-D-23-01354, titled: “A novel duplex qualitative real-time PCR assay for the detection and differentiation of *Plasmodium ovale curtisi* and *Plasmodium ovale wallikeri*” submitted to PLOS Neglected Tropical Diseases.**

**The authors describe a newly developed duplex real-time PCR assay for the detection and identification of the *Plasmodium ovale* spp. It is a further development of their previous work Potlapalli et al 2023. They detailed their methodology for the selection of the targets, the development and optimization of the assay, the evaluation of its performance on controls, its validation on a set of field samples, and then its application to study the epidemiology of the *Plasmodium ovale* spp. among symptomatic patients in 3 regions of the Democratic Republic of Congo. The manuscript is sounded, well developed and nicely written presenting all the expected steps for such topic.**

**While this tool will remain of little interest for the Medical doctors, who are still considering, managing and treating both species as one sole entity; this new assay will be useful for the Malariologists and the Malaria elimination program Managers to study the *P. ovale* spp. that are sympatric, wide spread, co-circulating in the endemic areas and widely overlooked. I am glad and would like to thank the authors for their contributing work on these neglected parasites.**

**Although a couple of points, listed below might need some clarifications / amendments to enhance the manuscript:**

1. **Regarding the nomenclature of the *P. ovale* spp., just recently, a modification in the taxonomy of these species have been proposed (Snounou et al 2024). This clarifies and simplifies the situation and should be adopted throughout the manuscript.**

**Response:** Thanks for this suggestion. Snounou et al. proposed new nomenclature of the *P. ovale* spp. (Snounou et al., 2024), whereas Šlapeta J et al. (Šlapeta J et al., 2024) raised objections to these new names. Due to the inconsistencies in the nomenclature of the *P. ovale* spp., we now use “*P. ovale curtisi*” and “*P. ovale wallikeri*” throughout the revised manuscript. Additionally, we acknowledge the new names proposed by Snounou et al. in the Introduction (see Introduction, L99-101).

2. **L24 and L25: “*P. ovale*”, *Plasmodium* should be written in full as first mention.**

**Response:** Updated as requested (see Abstract, L24).

3. **Throughout the manuscript: the use of italic or not for the mentions of “non-ovale” and “non-*falciparum*” should be harmonized.**

**Response:** Thanks for the comments. We have attempted to properly italicize species names (but not clinical diagnoses such as “non-ovale malaria” or “non-falciparum malaria”) throughout the revised manuscript.

4. **L129-133: The *Poc* and *Pow* field samples used as controls were “well characterized”, using the earlier protocol proposed by the authors [9], why not comparing this new assay vs their previous one for Poc / Pow distinction and on their practicality and operation to highlight pros and cons?**

**Response:** Thanks for this suggestion. Unfortunately, not all samples had sufficient DNA to perform the suggested head-to-head testing (or sequencing needed to resolve discordances).

5. **L163: “species” no italic**

**Response:** Thanks for the comments. We revised it in the revised manuscript (see Materials and Methods, L220).

6. **L167-169: “Samples with ct values lower than 45 in duplicate…” this positivity cut-off sound not compatible with the number of cycles of the PCR which is also 45 (L233).**

**Response:** Thanks for the comments. We recommend using 40 cycles for *P. ovale curtisi* singleplex assay and 45 cycles for *P. ovale wallikeri* singleplex assay and the duplex assay (see Results, L307-308 and L319-320).

7. **L233-234: please precise on which equipment was the PCR done, based on the Fig 1, once can guess QuanStudio 6?**

**Response**: Thanks for the comments. We used the the BioRad CFX384 Touch Real-Time PCR Detection System for assay development and sample detection. We have added this in the revised manuscript (see Materials and Methods, L196-197) and revised Figure 1.

8. **An Internal Control, either multiplexed in or run in parallel of the developed PCR, would be useful to assess inhibition and/or DNA degradation as well as PCR efficiency. This could also contribute to understand the difference into the sensitivity of the assays.**

**Response:** We agree with the reviewer that this would improve the assay; however, it would also increase complexity and cost. We did not include internal control in this study but did include negative- and positive-control standards (serially diluted *P. ovale curtisi* and *P. ovale wallikeri* plasmid DNA solution) in each qPCR run (see Materials and Methods, L198-200). The Ct values of the positive standards were similar between different runs.

9. **L276-294 and Fig 4: conversely to what is advocated across rest of the manuscript, in the** **epidemiological study of symptomatic malaria cases due to *P. ovale* in 3 regions of DRC, the authors analysed and presented their results on *P. ovale* as a whole entity while being able to discriminate *Poc* and *Pow*. This appears defeating the rest of the manuscript and it would sound better to mention that there was no difference between *Poc* and *Pow* in relation to the epidemiological parameter measured instead of looping both together.**

**Response:** Thanks for the comments. We have removed the epidemiological analysis from the revised manuscript (Materials and Methods, L228-251; Results, L367-390; Discussion, L455-470).

10. **L328-329: please check and correct this sentence, actually the 18S rRNA are roughly present in the same range of copy number per genome than the target used here in the duplex PCR, this might explain the similar LODs mentioned by the authors (L330-331). Please also amend accordingly below.**

**Response:** The 18S rRNA gene usually occurs in 5-7 copies per genome (Mercereau-Puijalon, et al. 2002). The copy number of our new targets for *P. ovale curtisi* and *P. ovale wallikeri* detection are 9 and 8 per genome. We revised this sentence as suggested (see Discussion, L434-439).

11. **L340: reference [44] is unrelated here.**

**Response:** We deleted this reference (Discussion, L452).

12. **L358: “higher” instead of “high”; interestingly in [47] they reported a difference in the distribution of *Poc* between urban and rural areas which was not observed for *Pow*… any similar findings here?**

**Response:** The epidemiological analysis was removed from the manuscript as noted above.

13. **L386: “eradication strategies” sounds somehow utopic, the best that could be achieved will be elimination.**

**Response:** Thanks for the comments. We used “elimination” in the revised manuscript (see Discussion, L502).

14. **As a separate though, from a quick glance at the primers and probes sequences, there is likely a substantial gap in the Tm between the *Poc* and *Pow* assay. The authors partly compensated this point during optimization by adjusting the** **concentration of the different components. But were the assays** **optimized in singleplex before being combined in duplex? This might give some indications to explain the difference into the assay sensitivity.**

**Response:** Thanks for the comments. We developed and optimized singleplex assays for *P. ovale curtisi* and *P. ovale wallikeri* before optimizing the duplex assay. We now include more information about the singleplex assays. The 95% confidence lower limits of detection of singleplex *P. ovale curtisi* and *P. ovale wallikeri* assays were 3.6 and 25.9 parasite genome equivalents/µl, respectively. The duplex assay has similar 95% confidence lower limits of detection as the singleplex assays, with 95% confidence lower limits of detection for *P. ovale curtisi* and *P. ovale wallikeri* as 4.2 and 41.2 parasite genome equivalents/µl, respectively. In the revised manuscript, we added details about singleplex assay development and their limits of detection (see Materials and Methods, L186-191 and L202-205; Results, L293-308 and L322-324).

15. **Another point to discuss for perspective could be the possibility to convert this assay into a dPCR assay to compensate the difference in sensitivity.**

**Response:** Thanks for this suggestion. We will consider this for future development, though have concerns about quantification due to losses in PCR efficiency caused by the short distances between *P. ovale curtisi* targets and between *P. ovale wallikeri* targets (Results, L288-289).

16. **Last, acknowledging the existence of some simian *ovale*-like parasites reservoir, the usefulness and interest of this new assay could be further discussed.**

**Response:** Interesting comment. Our assay was developed using human *P. ovale curtisi* and *P. ovale wallikeri* references. We did not find any simian *ovale*-like parasite genome references in NCBI database.

17. **Altogether, it is an interesting manuscript that I would recommend for publication into the PLoS NTD provided some minor amendments are performed.**

**Response:** Thank you for your encouraging comments. We believe the manuscript has been strengthened by all of the reviewers’ comments.

18. **Line 73 Reference 1 is not adequate in this context.**

**Response:** Thanks for the comments. We added more references to indicate the circulation of neglected *Plasmodium* species in different countries, including Cameroon, Nigeria, and Kenya (see Introduction, L96).

19. **Line 316/Line 322 LSA3 might be non-essential for blood-stage parasites, but it is** **essential for hepatic parasites. Furthermore, there is no evidence that LSA3 is involved in antigenic variation.**

**Response:** We have revised this sentence (see Discussion, L419-421).

20. **Line 331 In reference 18 the cut-off of 50 plasmid copies was established for a Ct of 38 or less.**

**Response:** Thanks for the comments. We have revised this sentence (Discussion, L436-439).

21. **A correction to the naming of the two species has been recently made (Snounou et al. Trends in Parasitology 2004 40:21), and it would be suitable to adopt the terms *P. ovalecurtisi* and *P. ovalewallikeri* in a revised manuscript.**

**Response:** Thanks for the comments. Snounou et al. proposed new nomenclature of the *P. ovale* spp. (Snounou et al., 2024), whereas Šlapeta J et al (Šlapeta J et al., 2024) raised objections to these new names. Due to the inconsistencies in the nomenclature of the *P. ovale* spp., we use “*P. ovale curtisi*” and “*P. ovale wallikeri*” throughout the revised manuscript. Additionally, we acknowledge the new names proposed by Snounou et al. in the Introduction (see Introduction, L99-101).

**Reviewer #3:**

**The authors have mined the genome of the two P. ovale species and selected in each a segment of DNA that is repeated in the genome and designed sets of oligonucleotide primers and corresponding probes that were then tested in real-time PCR assays for specificity and sensitivity. One set for each species that was identified as giving the optimal results was then tested using a selected set of P. ovale-positive samples (identified in a previous study), and this new protocol is advocated to perform better/to be more practical than others and therefore to serve for high throughput surveys in endemic countries. The authors then derived some epidemiological conclusions from the data obtained from these samples.**

**Major Comments**

1. **Whereas the approach of the authors (targeting internally repeated short segments) is original for *P. ovale* and the methodology and data are sound, the main thrust of this manuscript is that their methodology (duplex real-time PCR) is better suited than all other published methods to date for investigations on the epidemiology/biology of these parasite species, with the main supporting argument is that it is less time-consuming (fewer steps between sample collection and assay results). However, many factors (some quoted by the authors and others not) trump this minor advantage. First, the ten-fold lower sensitivity of detection for one species (Pow) as compared to the other (Poc) is a major limitation that would bias data from all field surveys, because a) it is well-known that *P. ovale* infections have low parasite densities past the primary peak that often persist for long durations, and b) low parasite densities, especially in** **mixed and asymptomatic infections, are only detected when highly sensitive molecular assays capable of detecting very low parasite levels (1 P/µl or less).**

**Response:** We agree with the reviewer that the lower sensitivity of the duplex assay for *P. ovale wallikeri* is an important limitation. In response to these and other reviewer comments, we have revised the manuscript to better highlight this limitation (see Discussion, L471-474). Nonetheless, our assay targets are distinct from those used in prior assays, exhibiting high specificity for *P. ovale curtisi* and *P. ovale wallikeri* (see Discussion, L432-434). We also include additional data for singleplex versions of the *P. ovale curtisi* and *P. ovale wallikeri* assays, respectively (see Materials and Methods, L186-191 and L202-205; Results, L293-308 and L322-324). Our objective was to enable end users to choose the assay format best suited to their needs. For an analysis focused on lower-density *P. ovale curtisi* infections, the *P. ovale curtisi* singleplex or duplex assay could be used. For an analysis seeking to whole-genome sequence higher density infections, the duplex assay could be used to identify *P. ovale curtisi* or *P. ovale wallikeri* samples. We’ve tried to provide clear use-cases in the revised discussion so that end-users can choose the approach best suited to their needs (see Discussion, L407-416 and L448-453).

Regarding mixed infections, the duplex assay successfully detected *P. ovale curtisi* and *P. ovale wallikeri* infections as part of mixed infection with other *Plasmodium* spp. Among the 55 field samples included in this study, 35 samples were *P. ovale* and *P. falciparum* mixed infections; 1 sample was a *P.ovale* and *P. malariae* mixed infection; 6 samples were *P. ovale*, *P. malariae*, and *P. falciparum* infections (see Fig 3). The parasite densities of the *P. falciparum* infections ranged from 0.01 parasites/µl to 23,647 parasites/µl, and the parasite densities of *P. malariae* infections ranged from 11 parasites/µl to 1,062 parasites/µl.

2. **It is difficult from the description given to work out the actual sensitivity that was obtained. The values (4,2 and 41,2 P/µL for Poc and Pow, respectively) quoted were from the Probit analysis using diluted plasmids. There is no indication as to the Plasmodium sequence that these plasmids contained. Moreover, it is not clear to what volume of blood the 3 µL DNA template obtained from Chelex-purified filter papers corresponds. Assuming 10 µL per filter spot, and 100 µL of DNA solution post-Chelex extraction, the 3 µL would correspond to 0,3 µL of blood. Is this the case? Furthermore, were the DNA templates used obtained from leukodepleted blood?**

**Response:** See response to Reviewer 1 comment #16 above. We have added additional details to make this clearer. We used dried blood spots containing approximately 70µl of whole blood for DNA extraction, eluted into 150µl final volume. Thus, 3µl DNA template is equivalent to 1.4µl of whole blood (see Materials and Methods, L176-179; Results, L299-300). The *P. ovale curtisi* and *P. ovale wallikeri* sequences contained in these plasmids are shown in Table S3. We also provide results of replicate testing for singleplex and duplex assays in the revised Supplementary Materials (Tables S4 and S5).

3. **One final point concerns the potential loss of sensitivity when the reaction is multiplexed, which is the case here. The authors should conduct experiments in which different proportions of DNA template from each species are mixed to assess whether the assay is capable of detecting a population of *Poc* or *Pow* when present as a minor proportion of the parasites (for instance in samples with high *P. falciparum* parasitaemias and only a few *P. ovale* per µL of blood).**

**Response:** We have reframed the manuscript to emphasize the strong performance of the *P. ovale curtisi* assay and better highlight limitations of the *P. ovale wallikeri* assay. During testing of field samples from the DRC, we confirmed detection of both *P. ovale curtisi* and *P. ovale wallikeri* in the presence of mixed infection with other *Plasmodium* spp (Fig 3). Adding detail to our response above. Among the 55 field samples included in this study, 35 samples were *P. ovale* and *P. falciparum* mixed infections; 1 sample was a *P.ovale* and *P. malariae* mixed infection; 6 samples were *P. ovale*, *P. malariae*, and *P. falciparum* infections. The parasite densities of the *P. falciparum* infections ranged from 0.01 parasites/µl to 23,647 parasites/µl, and the parasite density of *P. malariae* infections ranged from 11 parasites/µl to 1,062 parasites/µl. Among these mixed infections, we detected *P. ovale curtisi* DNA in mixed infections with high *P. falciparum* and *P. malariae* parasite densities (23,647 parasites/µl and 939 parasites/µl for *P. falciparum* and *P. malariae*, respectively). *P. ovale wallikeri* DNA was also detected in mixed infections with high *P. falciparum* and *P. malariae* parasite densities (8,927 parasites/µl and 1,062 parasites/µl for *P. falciparum* and *P. malariae*, respectively).

4. **There is little known concerning the global diversity of the repeats in the putative lsa3 gene and for the untranslated repeats on which the assay is based. Although it is likely that potential variations would not affect sensitivity, all the samples used to validate the assay were collected from the DRC and a few from Tanzania. It would have been very useful to include samples from West Africa and Oceania.**

**Response:** Thanks for the comments. As part of a separate project, our group recently used hybrid capture and selective whole genome amplification assays to whole-genome sequence 13 *P. ovale curtisi* samples from 4 countries (the DRC, Camerron, Tanzania, and Ethiopia) and 12 *P. ovale wallikeri* samples from 3 countries (the DRC, Tanzania, and Ethiopia). We queried these genomes (Carey-Ewend et al. Available via pre-print: <https://www.biorxiv.org/content/10.1101/2024.04.10.588912v1>), as well as the published *P. ovale curtisi* and *P. ovale wallikeri* genomes, and found only low level of variation in the targeted regions. The limited number of variants observed corresponded to sites for which our Poc and Pow primers already included “R” and Pow probe already included “Y” that are complementary. We did observe small differences in the copy number of the targets for *P. ovale curtisi* and *P. ovale wallikeri* varied from 8-9 and 7-8, respectively. Thus, available genomic data from multiple countries indicates (but of course doesn’t confirm definitively) that our assay is suitable for samples from other regions.

5. **Ultimately the authors have not provided sufficient evidence to support their claim that this novel method is superior to others for field investigations of the two P. ovale species. One might argue that the high rate of false negatives is a major disadvantage of the assay: 11 of the 55 P. ovale 5 samples used to validate the assay were not identified, i.e. a 20% failure rate, even if this was mainly in cases with low parasite burdens, A shorter processing time does not compensate for the lower sensitivity, and the techniques used still require a well-equipped laboratory.**

**Response:** Thanks for the comments. We have added a new supplementary table outlining the limits of detection and characteristics of published assays to-date (Table S1). We agree that the lower performance for *P. ovale wallikeri* is a limitation of the assay, but also note that existing assays have limitations of their own. We hope that the new assays described in our manuscript will be of value to the research community, both due to the strong performance of the *P. ovale curtisi* assay and the use of novel repetitive targets for *P. ovale curtisi* and *P. ovale wallikeri*. We have outlined potential use cases in the revised manuscript to make it clearer when/where these assays are most useful (see Discussion, 407-416, L432-434, and L448-453).

6. **The conclusions from the epidemiological analyses are at best speculative because they are based on a restricted number of samples from symptomatic cases collected from various locations in the DRC, and because of the bias in the detection of Pow. The authors acknowledge the limitations in the Discussion. I suggest that this section is omitted from the manuscript.**

**Response:** We have removed this section altogether, as recommended (Materials and Methods, L228-251; Results, L367-390; Discussion, L455-470).
